# Cigarette Smoke Modulates Expression of Human Rhinovirus-Induced Airway Epithelial Host Defense Genes

**DOI:** 10.1371/journal.pone.0040762

**Published:** 2012-07-12

**Authors:** David Proud, Magdalena H. Hudy, Shahina Wiehler, Raza S. Zaheer, Minaa A. Amin, Jonathan B. Pelikan, Claire E. Tacon, Tabitha O. Tonsaker, Brandie L. Walker, Cora Kooi, Suzanne L. Traves, Richard Leigh

**Affiliations:** 1 Airway Inflammation Research Group, Department of Physiology and Pharmacology, Snyder Institute for Chronic Diseases, University of Calgary Faculty of Medicine, Calgary, Alberta, Canada; 2 Airway Inflammation Research Group, Department of Medicine, Snyder Institute for Chronic Diseases, University of Calgary Faculty of Medicine, Calgary, Alberta Canada; University of Tübingen, Germany

## Abstract

Human rhinovirus (HRV) infections trigger acute exacerbations of chronic obstructive pulmonary disease (COPD) and asthma. The human airway epithelial cell is the primary site of HRV infection and responds to infection with altered expression of multiple genes, the products of which could regulate the outcome to infection. Cigarette smoking aggravates asthma symptoms, and is also the predominant risk factor for the development and progression of COPD. We, therefore, examined whether cigarette smoke extract (CSE) modulates viral responses by altering HRV-induced epithelial gene expression. Primary cultures of human bronchial epithelial cells were exposed to medium alone, CSE alone, purified HRV-16 alone or to HRV-16+ CSE. After 24 h, supernatants were collected and total cellular RNA was isolated. Gene array analysis was performed to examine mRNA expression. Additional experiments, using real-time RT-PCR, ELISA and/or western blotting, validated altered expression of selected gene products. CSE and HRV-16 each induced groups of genes that were largely independent of each other. When compared to gene expression in response to CSE alone, cells treated with HRV+CSE showed no obvious differences in CSE-induced gene expression. By contrast, compared to gene induction in response to HRV-16 alone, cells exposed to HRV+CSE showed marked suppression of expression of a number of HRV-induced genes associated with various functions, including antiviral defenses, inflammation, viral signaling and airway remodeling. These changes were not associated with altered expression of type I or type III interferons. Thus, CSE alters epithelial responses to HRV infection in a manner that may negatively impact antiviral and host defense outcomes.

## Introduction

Human rhinoviruses are not only the predominant cause of the common cold, but are also the major viral pathogens associated with acute exacerbations of asthma and chronic obstructive pulmonary disease (COPD) [1], [2]. In both the upper and lower airways, the primary site of human rhinovirus (HRV) infection is the airway epithelial cell [3], [4]. Because HRV infections do not result in overt epithelial cytotoxicity, it is generally assumed that virus-induced alterations in epithelial cell biology help to determine the clinical outcomes to infection in infected individuals [5]. In support of this, studies have demonstrated alterations in expression of a large number of genes in epithelial cells both when infected in tissue culture [6], [7], as well as in cells obtained directly during *in vivo* experimental HRV infections [8].

Cigarette smoking has profound effects on human health and is the major risk factor associated with the development and progression of COPD. Cigarette smokers experience more frequent and severe upper respiratory viral infections than non-smokers [9], [10]. In addition, approximately 25% of patients with asthma smoke, and such patients experience more severe symptoms, more frequent hospitalizations, and a worse quality of life than asthmatic patients who do not smoke [11]. In a recent study, subjects hospitalized for rhinovirus-induced exacerbations of asthma were significantly more likely to be current smokers [12].

**Table 1:** Primers and probes used for real-time RT-PCR analyses.

Gene	Forward Primer	Reverse Primer	Probe/Detection
**CXCL10**	gaaattattcctgcaagccaattt	tcacccttctttttcattgtagca	tccacgtgttgagatca
**CCL5**	tctgcgctcctgcatctg	agtgggcgggcaatgtag	attcctcggacaccacaccctgctg
**ISG15**	gctgggacctgacggtga	tggagctgctcagggacac	atgctggcgggcaacgaattcc
**ISG56**	aagccctggagtactatgagcg	gcctaaggaccttgtctcacaga	ccctgagactggctgctgactttgagaa
**Viperin**	cctgcttggtgcctgaatct	gcgcatatattcatccagaataagg	accagaagatgaaagact
**RIG-I**	caggatttgtaaagccctgttttt	cactgataatgagggcatcattatattt	tacacttcacatttgcg
**Mda-5**	tggtcgagccagagctgat	actcctgaaccactgtgagcaa	agagcacctacgtcctg
**STAT-1**	gcccaatgcttgcttggat	gctgcagactctccgcaact	agctgcagaactggtt
**EPSTI1**	tgcatacaccttgatagcaccaa	tcctgctccgcaattctttg	SYBR green

All sequences are given in the 5′ to 3′ direction. All Taqman probes were FAM-MGB labeled.

Although there is abundant evidence that cigarette smoking is associated with an increased prevalence of, and more severe outcomes from, respiratory viral infections, the mechanisms responsible for this have not been fully defined. It has been suggested, however, that cigarette smoke modulates immune and inflammatory responses to pathogens [13]. We, and others, have recently demonstrated that cigarette smoke extract (CSE) alters the profile of epithelial chemokine production in response to HRV infections in a manner that would be expected to impact innate immune responses [14], [15]. Thus far, however, there has been no comprehensive evaluation of the effects of acute CSE exposure on epithelial responses to HRV infection. The current studies were performed using gene array analysis to examine how CSE exposure modifies HRV-induced epithelial gene expression, with additional studies using real-time RT-PCR and protein analyses to validate selected observations from the array analysis.

## Materials and Methods

### Materials

The following reagents were purchased from the indicated suppliers: bronchial epithelial cell basal medium (BEBM) and additives to create serum-free bronchial epithelial cell growth medium (BEGM) (Lonza, Walkersville, MD); WI-38 cells were purchased from the American Type Culture Collection (Manassas, VA); HBSS, TRIzol reagent, and FBS (Invitrogen, Burlington, ON, Canada); DNase I (Ambion, Austin, TX); TaqMan Master Mix, 20X GAPDH, RNase inhibitor and reverse transcriptase (Applied Biosystems, Foster City, CA); antibodies to signal transducer and activator of transcription 1 (STAT1), retinoic acid inducible gene-I (RIG-I), and interferon inducible gene of 15 kDa (ISG15) were from Cell Signaling Technology, Inc (Danvers, MA); antibody to ISG56 (IFIT1) was from Abcam (Cambridge, MA); antibody to melanoma differentiation-associated gene 5 (mda-5) was from Alexis Biochemicals (San Diego, CA), antibody to viperin was from Enzo Life Sciences (Plymouth Meeting, PA); antibody to epithelial stromal interaction 1 (EPSTI1) was from Abnova (Walnut, CA)**.** All other chemicals were purchased from Sigma-Aldrich (Oakville, ON, Canada).

**Table 2:** Genes Most Significantly Induced after 24 h Exposure of Human Bronchial Epithelial Cells to Cigarette Smoke Extract (CSE).

Gene	Description	Fold Induction
**Metabolism/Redox Pathways:**		
CYP1A1	cytochrome p450, family 1, subfamily A, polypeptide 1	**116**
CYP1B1	cytochrome p450, family 1, subfamily B, polypeptide 1	**10.3**
NQO1	NADPH dehydrogenase, quinone 1	**7.3**
GPX2	glutathione peroxidase 2	**5.5**
ALDH3A1	aldehyde dehydrogenase 3 family, member A1	**4.8**
G6PB	glucose-6-phosphate dehydrogenase	**3.6**
TXNRD1	thiodoxin reductase 1	**3.5**
AKR1C2	aldo-keto reductase, family 1, member C2	**2.9**
AKR1C3	aldo-keto reductase, family 1, member C3	**2.6**
**Heme metabolism/iron binding:**		
HMOX1	heme oxygenase (decycling) 1	**4.4**
PIR	pirin (iron-binding nuclear protein)	**2.4**
FTH1	ferritin, heavy polypeptide 1	**2.3**

### Epithelial Cell Cultures

Normal human lungs from non-smoking individuals that were not used for transplant were obtained from a non-profit tissue retrieval service (International Institute for the Advancement of Medicine, Edison, NJ). All donors had negative serologies for HIV1/2, HTLV1/2, hepatitis A, B and C, and syphilis. In the current studies, cells derived from lungs of 4 individual donors were used for gene array analysis (3 male, age range 25–63 years old). Cells derived from an additional 12 individual donors (10 male, age range 17–57 years old) were used for additional validation experiments. All of these lung donors died from cerebrovascular causes or from accidental head trauma. Primary human bronchial epithelial cells (HBE) were obtained by protease digestion of dissected airways as previously described [16]. In brief, airways were dissected from lungs and incubated in F12 medium containing pronase (0.1%) and gentamycin (50 µg/ml) at 4°C for approximately 36 h. After this incubation period, tissue was placed in F12 medium supplemented with 10% FBS, dissected longitudinally, and epithelial cells were dislodged from the airway surface by jets of medium delivered from a 3 ml syringe. Cells were centrifuged at 350×*g* for 8 min, resuspended at 0.5×10^6^ per ml in freezing medium, frozen, and stored in a liquid nitrogen freezer until use. Cells obtained using these methods were confirmed to be epithelial cells by cytokeratin staining [16]. HBE were grown in BEGM and incubated at 37°C in 5% CO_2_. Cells were cultured overnight in BEGM from which hydrocortisone was removed before stimulation and this medium was used for all experiments.

### Ethics Statement

As noted above, human lungs were obtained from a non-profit tissue retrieval service. Individual hospital sites obtained consent from responsible next of kin for tissues to be used for either transplant or for research. The retrieval service coordinated shipments of lungs. These are received without any unique patient identifiers. Thus, as “end users” we had no input on patient care, nor any information on patient identity. Approval to use recovered organs for these studies was obtained from the Conjoint Health Research Ethics Board of the University of Calgary.

**Table 3:** Genes Most Significantly Induced 24 h post HRV-16 Infection of Human Bronchial Epithelial Cells Plus selected others (italics), and Effects of Simultaneous Exposure to Cigarette Smoke Extract (CSE).

Gene	Description	Fold Induction	
		HRV-16	HRV-16+ CSE
**Chemokines:**			
CXCL10	chemokine (C-X-C motif) ligand 10	**540**	**65**
CCL5	chemokine (C-C motif) ligand 5	**135**	**52**
CXCL11	chemokine (C-X-C motif) ligand 11	**100**	**22**
**Potential Antiviral:**			
IFIT1	interferon-induced protein with tetratricopeptide repeats 1 (ISG56)	**507**	**170**
IFI44L	interferon-induced protein 44-like	**135**	**49**
IFIT3	interferon-induced protein with tetratricopeptide repeats 3	**100**	**30**
RSAD2	viperin	**90**	**22**
OASL	oligoadenylate synthase like	**90**	**30**
MX1	myxovirus (influenza virus) resistance 1	**80**	**28**
IFI6	interferon, alpha-inducible protein 6	**79**	**26**
IFI44	interferon-induced protein 44	**73**	**32**
HERC5	hect domain and RLD-5	**71**	**18**
IFIT2	interferon-induced protein with tetratricopeptide repeats 1 (ISG54)	**57**	**13**
ISG15	interferon induced protein of 15 kDa	**46**	**17**
OAS2	2′,5′-oligoadenylate synthase 2	**30**	**12**
*IFNB1*	*interferon beta 1*	***20***	***8***
*IL28A*	*interleukin 28A (interferon, lambda 2)*	***13***	***10***
*IL29*	*interleukin 29 (interferon, lambda 1)*	***9***	***4***
**Signaling:**			
DDX58	retinoic acid inducible protein-I (RIG-I)	**32**	**15**
IFIH1	melanoma differentiation associated gene-5 (mda-5)	**23**	**10**
STAT-1	signal transducer and activator of transcription-1	**16**	**8**
IRF-7	Interferon regulatory factor 7	**15**	**9**
**Adhesion/Remodeling:**			
EPSTI1	epithelial stromal interaction 1	**33**	**11**
*FGF2*	*fibroblast growth factor 2 (basic)*	***4***	***1.7***
*AREG*	*amphiregulin*	***4***	***4***
*ICAM-1*	*intercellular adhesion molecule 1*	***3***	***3***

### Preparation of Purified HRV and of CSE

HRV-16 was propagated in WI-38 cells and purified by centrifugation over sucrose as previously described [17], [18]. Viral titers were determined as previously described [18].

CSE was freshly prepared, as described [14], by a minor modification of previously published methods [19]. In brief, crude CSE was generated by bubbling one research grade cigarette (3R4F, College of Agriculture Reference Cigarette Program, University of Kentucky) per 4 ml of BEGM without hydrocortisone at a rate of 5 minutes per cigarette using a syringe apparatus. The crude CSE was filtered through a 0.22 µm filter and subsequently adjusted with medium to an absorbance of 0.15 at 320 nm. This solution was taken as 100% CSE. All epithelial exposures in the current study were done using 50% CSE. We have previously shown that this concentration of CSE does not affect replication of HRV during the first 24 h incubation, nor does it cause any epithelial cell toxicity [14].

**Table 4:** Genes Most Significantly Down-Regulated 24 h post HRV-16 Infection of Human Bronchial Epithelial Cells, and Effects of Simultaneous Exposure to Cigarette Smoke Extract (CSE).

Gene	Description	Fold Induction	
		HRV-16	HRV-16+ CSE
DLGAP1	discs, large (Drosophila) homolog associated protein 1	**−5.0**	**−1.6**
DHFRL1	dihydrofolate reductase-like 1	**−4.8**	**−1.4**
RAP80	receptor associated protein 80	**−4.5**	**−6.8**
KIAA0992	palladin	**−3.6**	**−1.2**
FLJ14011	zinc finger protein 667	**−3.5**	**−1.1**
HKDC1	hexokinase domain containing 1	**−3.5**	**−1.4**
CSPG4	chondroitin sulfate proteoglycan 4	**−3.5**	**−3.3**
CYorf15A	chromosome Y open reading frame 15A	**−3.4**	**−1.6**
GUCA1A	guanylate cyclase activator 1A	**−3.4**	**−1.2**
PMS1	PMS1 postmeoitic segregation increased 1	**−3.4**	**−3.0**
CLDN18	claudin 18	**−3.1**	**−1.8**
KIF15	kinesin family member 15	**−3.1**	**−4.2**
ZNF483	zinc finger protein 483	**−3.1**	**−2.5**
C20orf19	chromosome 20 open reading frame 19	**−3.1**	**−1.4**
PPARGC1A	peroxisome proliferator-activated receptor gamma, coactivator 1	**−3.0**	**−2.2**

### Viral Infection and Stimulation of Epithelial Cells

HBE were exposed to medium, HRV-16 alone at 10^5.5^ 50% tissue culture-infective dose (TCID_50_) U/ml (multiplicity of infection (MOI) of ∼1), 50% CSE alone, or HRV-16+ CSE. It was not financially feasible to perform array analyses on multiple time points for all treatments. We chose to incubate HBE with stimuli at 34°C in 5% CO_2_ for 24 h, both because this was the time that had been used in earlier HRV array experiments with cultured cells [6], [7] and would therefore best permit comparison of our data with other studies, and because we, and others, have previously shown CSE-induced modulation of viral induction of some genes at this time point [14], [15]. After the 24 h incubation, supernatants were harvested, and total cellular RNA was isolated using Trizol reagent. In additional experiments, total cell lysates were prepared and used for western blotting.

### Gene Array Analysis

Preparation of samples and gene array analyses were performed by Expression Analysis, Inc (Durham, NC). Purified RNA was quantified using a Nanodrop spectrophotometer and quality was determined using the Agilent (Santa Clara, CA) 2100 Lab-on-a-Chip System. Purified RNA was converted to GeneChip target using Affymetrix GeneChip 3′ IVT express kit (Affymetrix, Santa Clara, CA). GeneChip target was then hybridized to Affymetrix U133plus2.0 human GeneChips for analysis of over 47,000 transcripts and then washed, stained, and scanned using the protocol described by Affymetrix. Data from all genomics samples underwent rigorous quality control procedures to detect potential outliers due to processing, instrumentation, or other reasons. This process included examination of the GeneChip level Affymetrix QC metrics: Raw Q, Scaling Factor, Noise Average, Background Average, Percent Present, and Percent Absent. Microarray data, including all raw Affymetrix data, were submitted, in a format that complies with the Minimal Information About a MicroArray Experiment (MIAME) guidelines, to the Gene Expression Omnibus (http://www.ncbi.nlm.nih.gov/projects/geo/) and have been assigned the accession number GSE27973.

**Figure 1:**
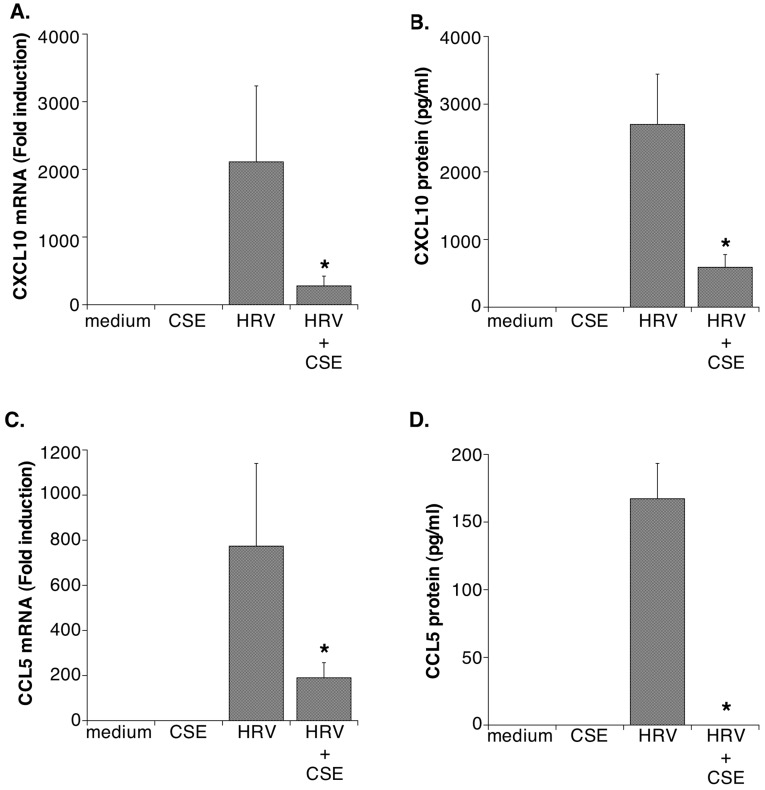
CSE suppresses HRV-induced expression of chemokines. HRV-induced expression of mRNA (Panel **A**) and protein (Panel **B**) for CXCL10, as well as mRNA (Panel **C**) and protein (Panel **D**) for CCL5 were significantly inhibited in the presence of CSE. Data are presented as mean ± SEM from 4–7 donors. Asterisks indicate significant inhibition for HRV+CSE compared to HRV alone.

### Real Time RT-PCR

Validation of increased mRNA expression for selected genes was performed by real time RT-PCR. For each sample, 400 ng of input RNA was reverse transcribed into cDNA, followed by PCR amplification. For some genes, this was performed using specific primers and probes for the gene of interest, while, for others, specific primers were used together with SYBR green to assess expression levels. For interferon (IFN)β, IL-29 (IFN-λ1) and IL28A (IFN-λ2) pre-designed “assays on demand” were used (Applied Biosystems, Streetsville, ON, Canada). In all cases, data were normalized for any minor variations in expression level of the housekeeping gene GAPDH. Data were expressed as fold induction using the ΔΔCt method. Primer and probe sequences for each gene examined are listed in **Table 1**.

**Figure 2 pone-0040762-g002:**
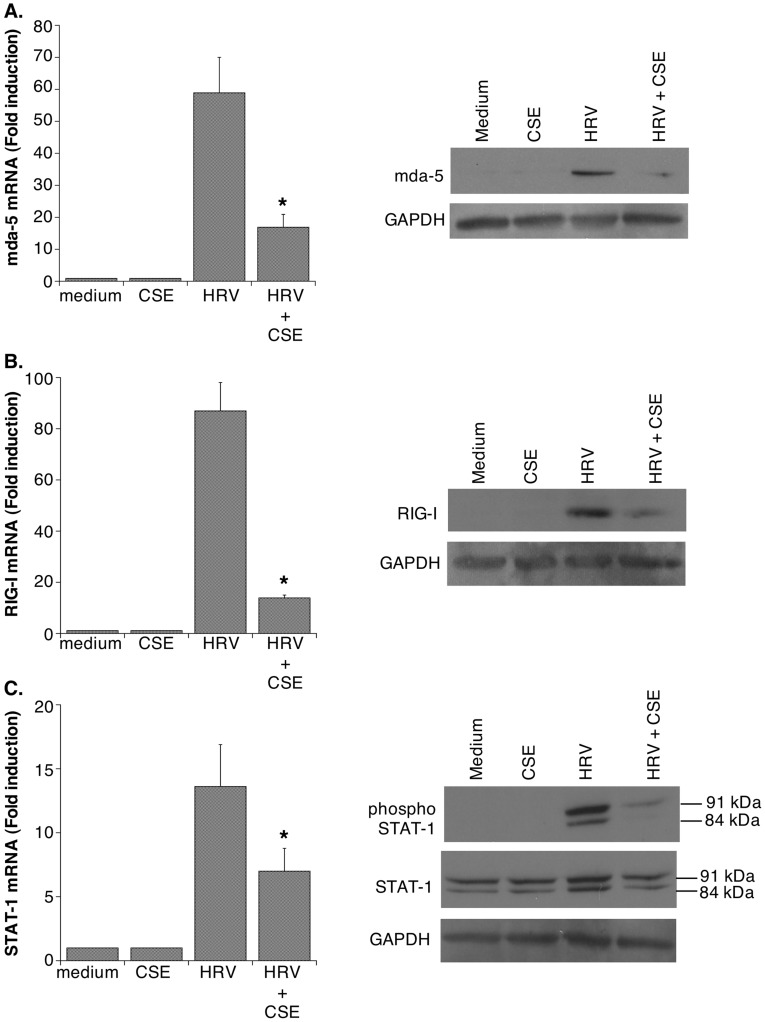
CSE suppresses HRV-induced expression of viral signaling molecules. The effects of CSE on HRV-induced expression of: **A.** mda-5, **B.** RIG-I and **C.** STAT-1, was examined. In each case, the left panel shows effects mRNA expression (mean ± SEM, n = 4–7). Asterisks indicate significant inhibition for HRV+CSE compared to HRV alone. The right panels show western blots (representative of n = 3 in each case) for each protein studied. In the case of STAT-1, phospho-STAT-1 was also examined. For all blots, glyceraldehyde 3-phosphate dehydrogenase (GAPDH) was assessed to ensure equal protein loading.

### Protein Assessments

For secreted chemokines and cytokines, cell supernatants were assayed by ELISA using matched antibody pairs according to the manufacturer’s protocol (R&D Systems, Minneapolis, MN). Minimum levels of detection were as follows: CXCL10 (23.5 pg/ml), CCL5 (31 pg/ml), Interferon (IFN)-λ1 (62.5 pg/ml). Unfortunately, with the best available antibodies, the assay for IFN-λ2 was the least sensitive, with a detection limit of 125 pg/ml. IFNβ was assayed using a Biosource ELISA kit sensitive to 2.5 pg/ml (Invitrogen, Burlington, ON, Canada). Cell associated proteins were assayed in total cell extracts that were subjected to SDS-PAGE and western blotting with appropriate antibodies, using previously described methods [20].

### Statistical Analysis

Comparisons of gene expression profiles between treatments were performed using two-group comparison analysis that incorporated permutation analysis for differential expression and accounted for multiple comparisons (Expression Analysis, Inc. Durham, NC). For all comparisons the statistical hypotheses were two-sided and p-values were reported. For assessments of mRNA and protein expression for individual genes of interest, normally distributed data were analyzed using one-way ANOVA with student Newman-Keuls post hoc analysis. Data that were not normally distributed were analyzed using Kruskal-Wallis ANOVA followed by Wilcoxon matched-pairs signed-rank test. Statistical hypotheses were two-sided and values of p<0.05 were considered significant.

**Figure 3 pone-0040762-g003:**
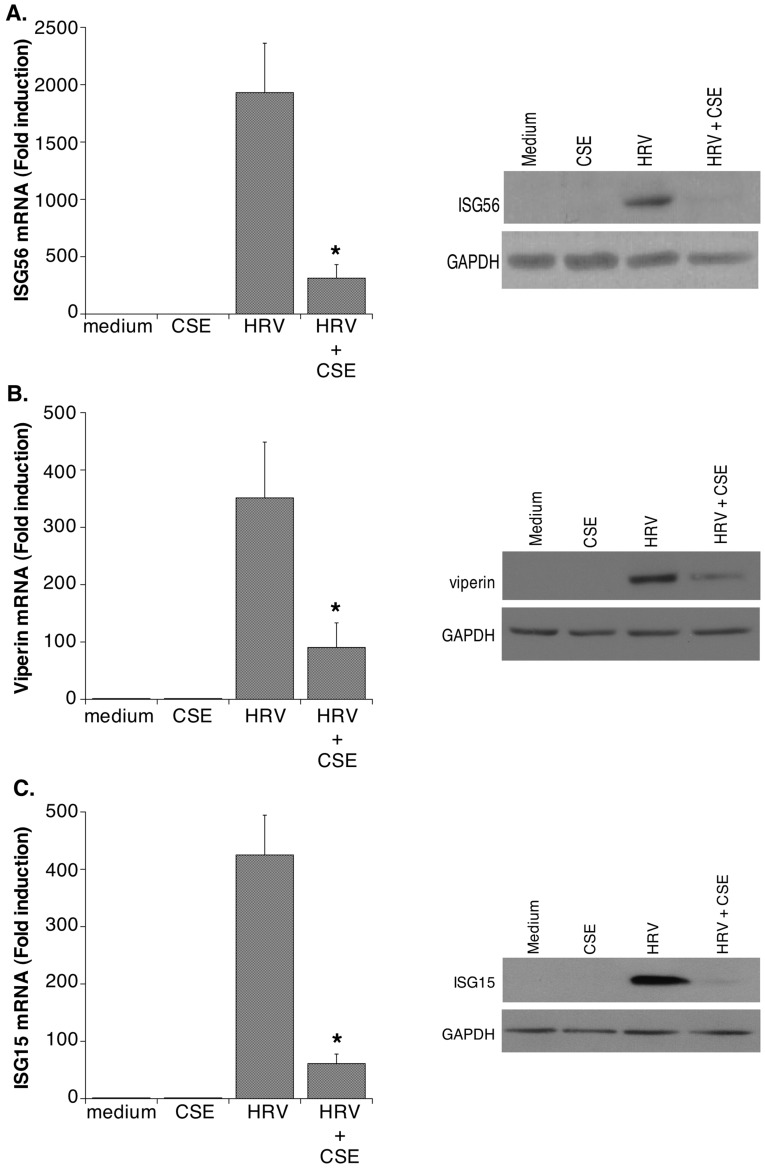
CSE suppresses HRV-induced expression of antiviral proteins. The effects of CSE on HRV-induced expression of: **A.** ISG56, **B.** Viperin and **C.** ISG15, was examined. In each case, the left panel shows effects mRNA expression (mean ± SEM, n = 4–7). Asterisks indicate significant inhibition for HRV+CSE compared to HRV alone. The right panels show western blots (representative of n = 3 in each case) for each protein studied. For all blots, GAPDH was assessed to ensure equal protein loading.

## Results

### Gene Array Analysis

#### a) CSE compared to medium

Using an arbitrary cut-off of a 2-fold change, expression of 5,547 transcripts increased by 2-fold or greater in cells exposed to CSE compared to cells exposed to medium, while 4,147 transcripts showed expression levels that were decreased to 0.5-fold or less compared to levels in cells exposed to medium. Approximately 2,500 of these expression changes were statistically significant. Of the most highly induced genes, many were associated with metabolism and/or redox pathways or with iron binding (**Table 2**).

**Figure 4:**
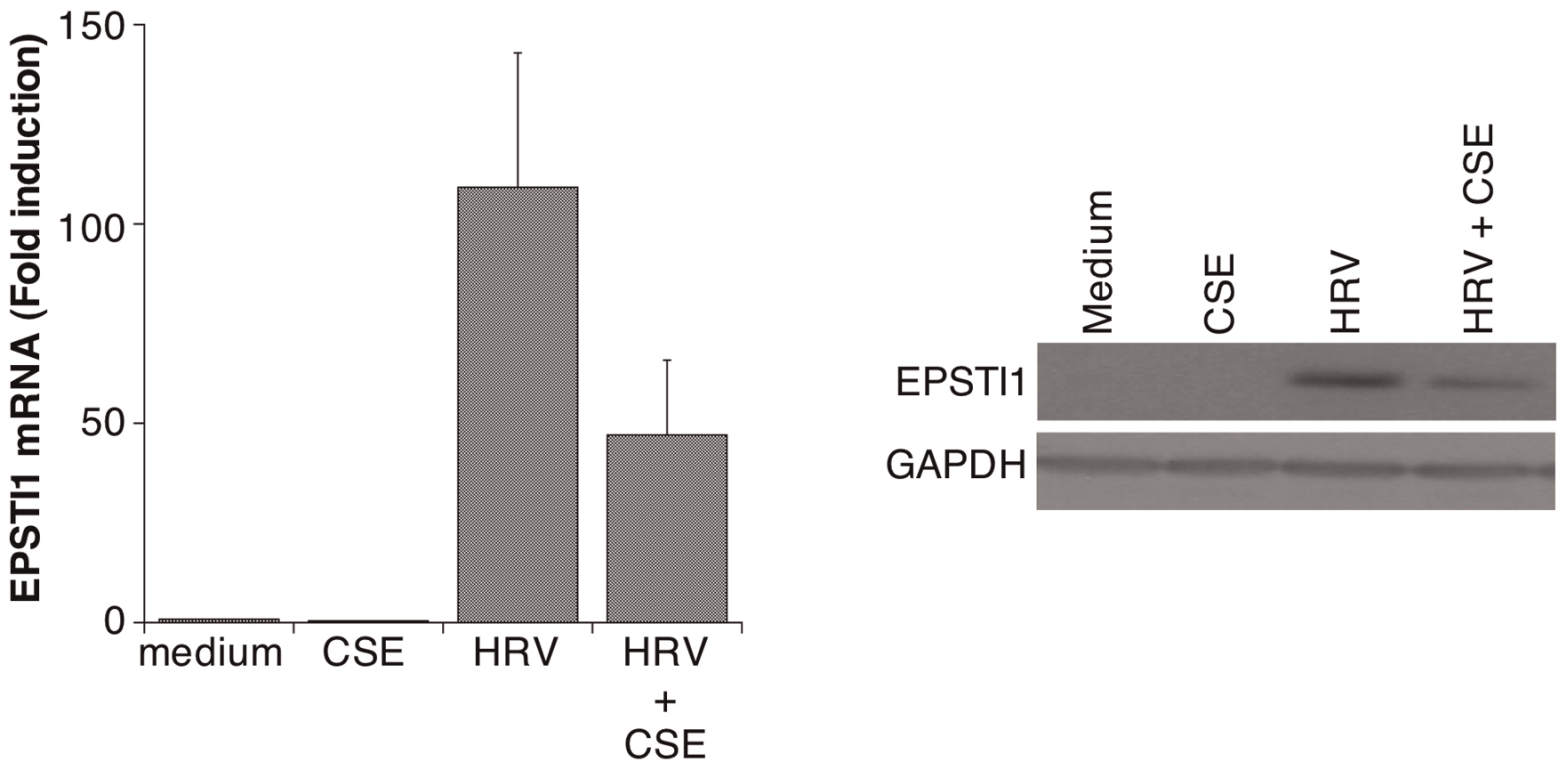
CSE suppresses HRV-induced expression of EPSTI1. The left panel shows effects mRNA expression (mean ± SEM, n = 5). The right panel shows a western blot (representative of n = 3) for EPSTI1 protein. GAPDH was assessed to ensure equal protein loading.

#### b) HRV-16 compared to medium

Expression of 6,494 transcripts increased by 2-fold or greater in cells infected with HRV-16 compared to cells exposed to medium, while 3,259 transcripts showed expression levels that were decreased to 0.5-fold or less compared to levels in cells exposed to medium. Over 3,000 of these changes in expression levels were statistically significant. As shown in **Table 3**, most of the most highly induced genes fell into discrete functional categories. These include chemokines of both the C-C and C-X-C families that regulate inflammatory cell recruitment and host defense, a variety of genes whose products have known or potential roles in antiviral defenses, genes associated with cytokine and viral signaling. EPSTI1 is a gene associated with adhesion processes and structural remodeling. EPSTI1 was not unique, and other genes that fall into this grouping were also significantly induced and are shown in italics in **Table 3**. Expression of type I and type II interferons is also shown, for reasons discussed below. There was no obvious overlap between genes highly induced upon exposure to CSE and those highly induced upon HRV infection. By contrast to the induced genes, there were no clear patterns in the genes that were down-regulated in cells upon HRV-16 infections. Examination of the 15 genes most significantly down-regulated (**Table 4**), for example, revealed no obvious functional groupings, other than the presence of two zinc-finger proteins.

#### c) Costimulation with HRV-16 and CSE

Interestingly, when cells were exposed to the combination of HRV-16 and CSE, the number of transcripts induced more than 2-fold relative to exposure to medium was less than either of the two stimuli alone. Only 4,988 transcripts were induced by more than 2-fold with 5,070 transcripts reduced to 0.5-fold or less. The combination of HRV-16 with CSE did not markedly affect the profile of highly expressed genes induced by CSE, with the genes shown in **Table 2** remaining as the most upregulated. The majority of the genes listed in **Table 2** were induced to comparable levels (Fold induction for CSE alone vs. CSE+HRV: CYP1A1, 116 vs. 67; CYP1B1, 10.3 vs. 8.5; NQO1, 7.3 vs. 6.1; GPX2, 5.5 vs. 4.2; MOX1, 4.4 vs. 4.0; PIR 2.4 vs. 2.1).

By contrast, when responses to HRV-16 alone were compared to those of the same cell donors exposed to the combination of HRV-16 and CSE, the most striking observation was that CSE markedly reduced HRV-induced expression of a substantial number of gene transcripts. This included expression of genes in each of the major categories of genes induced by HRV-infection (**Table 3**). It should be noted, however, that there was some selectivity to the genes reduced, as some genes from each category (e.g. IFNβ, interleukin 29 (IFN-λ1), IL-28A (IFN-λ2), IRF-7, amphiregulin and ICAM-1) were not significantly affected by the presence of CSE. Expression levels of the 15 genes most down-regulated upon HRV-16 infection (**Table 4**) were not significantly altered in the presence of CSE.

**Figure 5:**
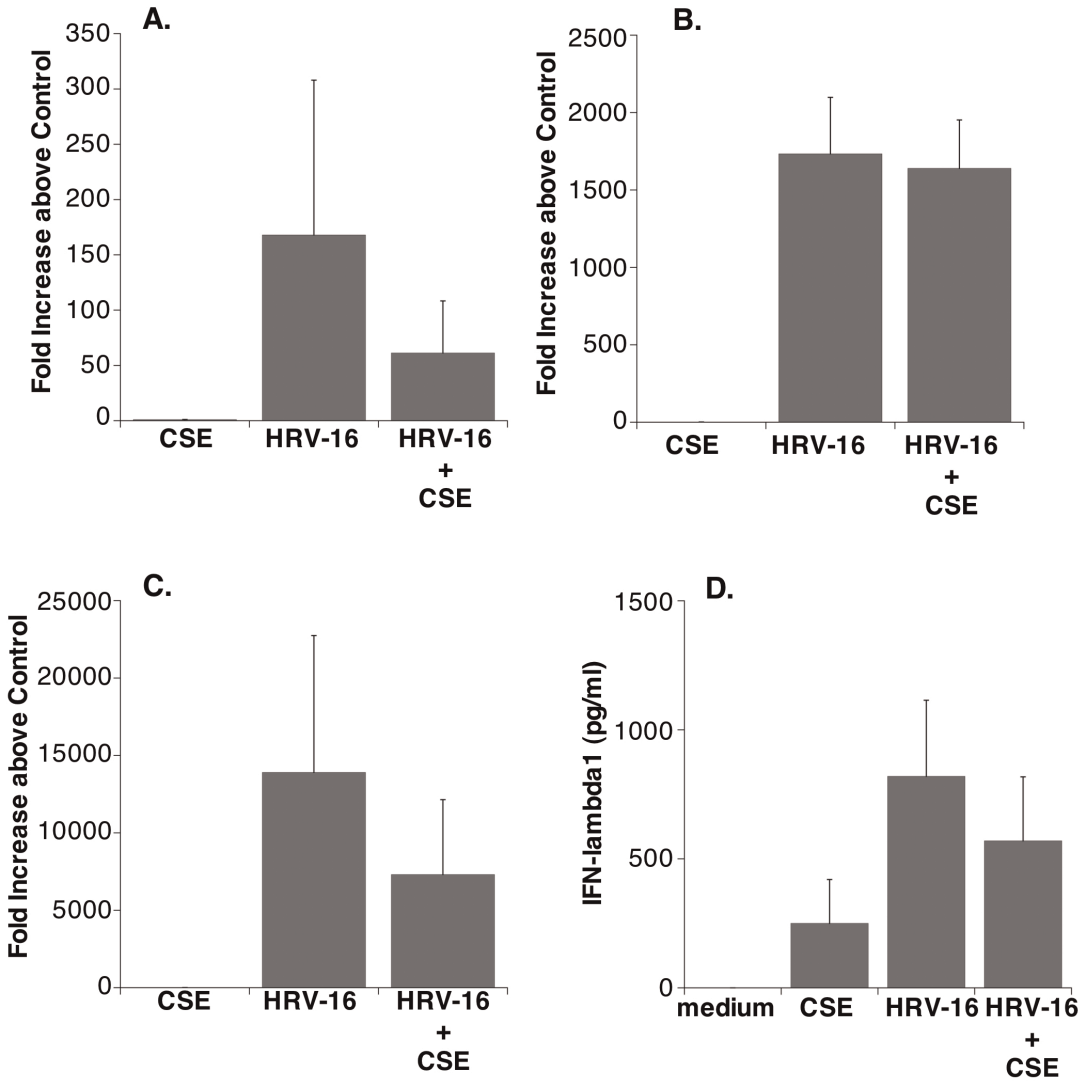
CSE does not alter HRV-induced expression of interferons. CSE did not significantly inhibit HRV-induced mRNA expression for : **A.** IFNβ, **B.** IFN-λ2 (IL28A) and **C.** IFN-λ1 (IL29). In each case, data are expresses as fold increase above medium control treatment (n = 5 in each case). Panel **D.** Shows protein expression for IFN-λ1 (IL29) expressed as pg/ml (n = 5). Data are presented as mean ± SEM in each case.

### Validation of Changes in Expression of Selected Genes and Proteins

To confirm the effects of CSE on HRV-induced expression of genes in a more quantitative fashion, we selected several genes from **Table 3** that were reduced at least 2-fold in the presence of CSE. Alterations in mRNA expression were rigorously assessed using real-time RT-PCR. Because altered gene expression may not always directly correlate with altered protein expression, we also monitored protein expression using ELISA or western blotting, as appropriate. As previously reported [14], [15], HRV-induced expression of both mRNA and protein for CXCL10 and CCL5 was significantly reduced in the presence of CSE (**Figure 1**). RIG-I, mda-5 and STAT-1 have all been implicated in HRV-induced signaling. Real-time RT-PCR and western blotting not only confirmed an earlier report that that CSE reduces HRV-induced expression of STAT-1 [15], but extended these data to show for the first time that viral expression of RIG-I and mda-5 is also significantly suppressed by CSE (**Figure 2** **).** To our knowledge the effects of CSE on epithelial induction of antiviral genes has not been examined previously. Our gene array analysis suggested a broad-ranging suppression of antiviral genes. Using real-time RT-PCR and western blotting we confirmed that HRV-induced expression of mRNA and protein for ISG56, viperin and ISG15, 3 genes with known, or potential, antiviral activity against HRV, was suppressed by CSE (**Figure 3**). Finally, we showed that HRV-induced expression of mRNA and protein for EPSTI1, a gene linked to epithelial-mesenchymal transition and tissue remodeling, was also significantly inhibited by concurrent exposure to CSE (**Figure 4**).

### Modulation of HRV-induced Gene Expression by CSE does not Appear to be Mediated via Changes in Interferon Expression

A substantial number of the genes listed in **Table 3**, particularly those associated with antiviral and host defense functions, also can be induced by interferons (IFNs), and, indeed, are often referred to as “interferon stimulated genes” (ISG). Thus it was plausible that CSE-mediated reduction of HRV-induced expression of these genes was secondary to effects on interferon expression. Quantitative real-time RT-PCR, however, failed to demonstrate a significant reduction in HRV-induced expression of mRNA for either type I (IFNβ), or type III (IFN-λ1 or IFN-λ2) IFNs in the presence of CSE (**Figure 5A-C**), confirming observations from the gene array. At the level of protein expression, HRV-induced epithelial production of IFN-λ1 was readily detected, but this also was not significantly reduced in the presence of CSE (**Figure 5D**). Measurable levels of IFN-λ1 were only detected in responses to CSE alone in 2 of 5 experiments. Although there appeared to be release of IFN-λ2 in some cultures this could not be accurately quantified as levels were at, or below, the limit of detection (125 pg/ml) for the assay (not shown). HRV-induced release of IFNβ from primary epithelial cell cultures could not be detected in any experiment using an ELISA with a minimum detection limit of 2.5 pg/ml. This was not due to degradation of protein as recovery of 50 pg/ml of recombinant IFNβ added to samples was 92.4±2.6% (mean ± SEM; n = 7).

## Discussion

The goal of these studies was to determine if CSE altered epithelial responses to HRV infection in a manner that, if reproduced by cigarette smoking *in vivo*, may be expected to lead to worse clinical outcomes to infection. We showed that both CSE and HRV-16 alter epithelial gene expression, but that the patterns of genes affected by each stimulus were quite discrete with no obvious overlap.

Consistent with earlier reports [21], [22], [23], exposure of epithelial cells to CSE alone induced increased expression of numerous genes linked to redox pathways, cellular metabolism and iron binding, although it should be acknowledged that some of these genes have been linked to other cellular functions, including apoptosis. Interestingly, when cells were exposed to the combination of HRV and CSE, the genes highly induced by CSE alone were largely unaffected and the profile of most highly induced genes was not altered.

The pattern of genes induced when epithelial cells were infected with purified HRV-16 was quite discrete from those induced by CSE. Consistent with earlier reports and ontogeny analyses of epithelial responses upon HRV infection either *in vitro* or *in vivo* [6], [7], [8], a broad array of genes involved in numerous aspects of viral signaling, innate immunity, antiviral defenses and inflammatory processes, and adhesion/remodeling were induced. In general, the fold induction of genes observed upon HRV infection of epithelial cells *in vitro* was considerably higher than those observed in the previously reported array conducted on epithelial scrapings obtained during experimental HRV infection *in vivo*. This may relate to the more controlled situation *in vitro*, where all cells are in growth medium and were continuously exposed to a standard dose of HRV throughout the 24 h incubation period. By contrast, samples were obtained 48 h after experimental infection *in vivo*. It is known that such experimental HRV inoculations lead to patchy, localized infection of epithelial cells with HRV. As such, only a limited number of cells recovered in a nasal scraping will be infected, and this may be expected to vary considerably among individuals. In addition, responses will likely depend to a much greater degree upon the extent of infection and the variation in levels of viral replication and shedding among individuals. It should be noted, however, that despite different absolute levels of gene induction and minor changes in the order of gene induction, there was a high concordance of those genes that were most upregulated in the two systems. For example, all of the genes shown to be upregulated in Table 3 were also upregulated upon experimental HRV infection *in vivo*.

Although simultaneous exposure to HRV and CSE did not markedly alter the expression of the majority of genes highly induced by CSE alone, concurrent exposure markedly reduced expression of a substantial number of HRV-induced epithelial genes in each of the major categories shown in **Table 3**. Many of the genes suppressed are linked to innate immunity and host antiviral defense. If similar changes in epithelial responses occur *in vivo* as a consequence of cigarette smoking, this could help explain why smokers with lower airway disease experience more frequent and more severe disease exacerbations than non-smokers. Co-stimulation with HRV and CSE did not significantly alter expression levels of the genes most down-regulated by HRV.

To validate key changes observed on gene array, we selected examples of genes from each category in **Table 3** and confirmed, using real-time RT-PCR and ELISA or western blotting, that both mRNA and protein expression for each gene were reduced upon exposure to the combination of HRV and CSE compared to HRV infection alone (**Figures 1** **–** [Fig pone-0040762-g002] [Fig pone-0040762-g003] **4**). The attenuation of virus-induced expression of these gene products by CSE would be expected to have a deleterious effect on the pathogenesis of HRV-induced disease exacerbations.

Both CXCL10 and CCL5 are increased in airway secretions during HRV infections [24], [25], and can contribute to inflammatory cell recruitment. As previously reported [14], [15], viral induction of both of these chemokines was significantly inhibited by CSE. Increases in CXCL10 production during HRV infections correlate with increased infiltration of activated T lymphocytes and natural killer (NK) cells that would be expected to contribute to antiviral defenses [24]. Reduction of CXCL10 expression, therefore, would also negatively impact immune responses to HRV infection. In support of this, CXCL10-knockout mice have decreased ability to control viral infections, and show impaired T cell recruitment and activation, while CXCL10 transgenic mice show improved control of infection and enhanced NK cell responses [26], [27]. CCL5 recruits a number of cell types, including lymphocytes, eosinophils and monocytes [28]. Reduced CCL5 production by cigarette smoke would be expected to limit eosinophilia and lymphocyte recruitment.

STAT-1 has been linked to HRV-induced, and dsRNA-induced, signal transduction [6]. Consistent with an earlier report [15], we found that CSE suppressed viral induction and activation of STAT-1, which would be expected to limit downstream induction of host defense molecules. We have now extended these observations to show that HRV-induced expression of the cytoplasmic RNA helicases, RIG-I and mda-5 are also suppressed by CSE. RIG-I and mda-5 are receptors for viral double-stranded RNA (dsRNA), and are known to play a central role in antiviral innate immune responses to viral infection [29]. Thus, impaired induction of these molecules in the presence of CSE could significantly limit antiviral innate immunity.

We have previously shown that blocking viperin expression in HRV-infected epithelial cells leads to increased viral replication [8]. Thus, reduced viperin expression in the presence of CSE would be expected to have a similar effect. The specific roles of ISG15 and of ISG56 (IFIT1) in HRV infections remain to be determined, but they are known to have antiviral effects against a range of other viruses. ISG15 is an ubiquitin-like modifier that can be covalently bound to both host and viral proteins and modify their respective functions [30]. Similarly, ISG56 contains multiple tetratricopeptide repeat motifs that mediate a range of protein-protein interactions, resulting in a variety of effects on cellular and viral functions, including viral replication, signaling and initiation of translation [31]. The ability of CSE to attenuate expression not only of these three molecules, but also of numerous other molecules with direct antiviral effects at 24 h post infection, would imply that HRV replication should be enhanced in the presence of CSE, at least at times after these molecules would be induced. Consistent with this expectation, we and others found that CSE did not modulate HRV replication in epithelial cells during the first 24 h after viral exposure, indicating that altered gene expression seen after 24 h exposure to HRV in combination with CSE is not due to effects on replication [14], [15]. By contrast, however, it has recently been shown that HRV replication is increased from 24–48 h post infection in epithelial cells exposed to CSE [15], suggesting that the induction of antiviral molecules during the initial 24 h period can modulate subsequent viral replication.

A substantial number of the genes involved in host defense, as well as those with direct antiviral actions, whose expression is suppressed by CSE are often referred to as “interferon stimulated genes” (ISG), raising the possibility that the effects of CSE on expression of these genes could be due to actions on interferon production. Indeed, it has been suggested that impaired production of type I and type III interferons are inherent features of both asthma and COPD, and may play a role in HRV-induced exacerbations of these diseases [32], [33], [34]. This remains controversial, however, as others have failed to find altered epithelial expression of IFNs in asthma [7], [35], and it has been reported that epithelial cells from COPD patients actually show enhanced type III IFN expression in response to HRV infection [36]. In the current study, effects of CSE on expression of HRV-induced genes appeared to occur independently of effects on IFNs. At the level of mRNA expression, there was no significant reduction in expression of IFNβ, IFN-λ1 or IFN-λ2 in the presence of HRV+CSE when compared to HRV alone. Consistent with earlier reports [33], [36], we detected HRV-induced release of IFN-λ1 protein from epithelial cells, but this was also not significantly reduced in the presence of CSE. We were not able to accurately measure effects of CSE on HRV-induced IFN-λ2 production as the levels generated by virus alone were at or below the level of detection. However, as noted, HRV-induced mRNA expression of IFN-λ2 was not significantly altered by CSE. We could not detect release of IFNβ protein from HRV-16-infected epithelial cells, even though recombinant protein added to samples was readily recovered. There has been considerable controversy regarding whether HRV-infection induces release of IFNβ protein from epithelial cells. Although some investigators have reported detectable levels [6], [32], our data are consistent with other reports that have also been unable to detect release of IFNβ protein despite detecting mRNA expression [24], [36], [37]. The reasons for these variations are unclear. Taken as a whole, however, our data suggest that CSE-mediated reduction of HRV-induced expression of antiviral and host defense genes occurs independently of changes in expression of type I or Type III IFNs. This would be consistent with a recent study showing that viral induction of a wide range of ISG can readily occur in cells that are deficient in both type I and type III interferon signaling [38]. Additional studies will be needed to establish the mechanisms by which CSE suppresses HRV-induced expression of genes of interest.

We recognize that a limitation of our study is that acute, *in vitro* exposure of epithelial cells to CSE may not necessarily mimic responses to chronic cigarette smoking *in vivo*. Therefore, we have obtained regulatory approval to perform experimental HRV infections in otherwise healthy smokers and non-smokers to determine if similar reductions in antiviral/host defense responses to *in vivo* HRV infection are observed in epithelial cells from individuals who smoke.

In summary, we provide the first evidence that exposure of epithelial cells to cigarette smoke extract *in vitro* suppresses HRV-induced expression of a substantial number of epithelial genes that play a role in direct host antiviral defenses, as well as of molecules involved in the initiation and signaling of innate immune responses that contribute to antiviral immunity. If cigarette smoking induces similar changes in epithelial responses to HRV infection *in vivo* these data could provide, at least in part, a mechanistic basis for the more severe clinical outcomes observed in smokers.

## References

[1] Traves SL, Proud D (2007). Viral-associated exacerbations of asthma and COPD.. Curr Opin Pharmacol.

[2] Jackson DJ, Johnston SL (2010). The role of viruses in acute exacerbations of asthma.. J Allergy Clin Immunol.

[3] Papadopoulos NG, Bates PJ, Bardin PG, Papi A, Leir SH (2000). Rhinoviruses infect the lower airways.. J Infect Dis.

[4] Mosser AG, Brockman-Schneider R, Amineva S, Burchell L, Sedgewick JB (2002). Similar frequency of rhinovirus-infectable cells in upper and lower airway epithelium.. J Infect Dis.

[5] Leigh R, Proud D (2011). Modulation of epithelial biology by rhinovirus infection: role in inflammatory airway diseases.. Future Virol.

[6] Chen Y, Hamati E, Lee P-K, Lee W-M, Wachi S (2006). Rhinovirus induces airway epithelial gene expression through double-stranded RNA and IFN-dependent pathways.. Am J Respir Cell Mol Biol.

[7] Bochkov YA, Hanson KM, Keles S, Brockman-Schneider RA, Jarjour NN (2010). Rhinovirus-induced modulation of gene expression in bronchial epithelial cells from subjects with asthma.. Mucosal Immunol.

[8] Proud D, Turner RB, Winther B, Wiehler S, Tiesman JP (2008). Gene expression profiles during *in vivo* human rhinovirus infection: insights into the host response.. Am J Respir Crit Care Med.

[9] Arcavi L, Benowtiz NL (2004). Cigarette smoking and infection.. Arch Intern Med.

[10] Cohen S, Tyrrell DA, Russell MA, Jarvis MJ, Smith AP (1993). Smoking, alcohol consumption, and susceptibility to the common cold.. Am J Public Health.

[11] McLeish AC, Zvolensky MJ (2010). Asthma and cigarette smoking: a review of the empirical literature.. J Asthma.

[12] Venarske DL, Busse WW, Griffin MR, Gebretsadik T, Shintani AK (2006). The relationship of rhinovirus-associated asthma hospitalizations with inhaled corticosteroids and smoking.. J Infect Dis.

[13] Stämpfli MR, Anderson GP (2009). How cigarette smoke skews immune responses to promote infection, lung disease and cancer.. Nat Rev Immunol.

[14] Hudy MH, Traves SL, Wiehler S, Proud D (2010). Cigarette smoke modulates rhinovirus-induced airway epithelial chemokine production.. Eur Respir J.

[15] Eddleston J, Lee RU, Doerner AM, Herschbach J, Zuraw BL (2011). Cigarette smoke decreases the innate responses of epithelial cells to rhinovirus infection.. Am J Respir Cell Mol Biol.

[16] Churchill L, Chilton FH, Resau JH, Bascom R, Hubbard WC (1989). Cyclooxygenase metabolism of endogenous arachidonic acid by cultured human tracheal epithelial cells.. Am Rev Respir Dis.

[17] Gern JE, Dick EC, Lee WM, Murray S, Meyer K (1996). Rhinovirus enters but does not replicate inside monocytes and airway macrophages.. J Immunol.

[18] Sanders SP, Siekierski ES, Porter JD, Richards SM, Proud D (1998). Nitric oxide inhibits rhinovirus-induced cytokine production and viral replication in a human respiratory epithelial cell line.. J Virol.

[19] Wirtz HRW, Schmidt M (1996). Acute influence of cigarette smoke on secretion of pulmonary surfactant in rat alveolar type II cells in culture.. Eur Respir J.

[20] Wiehler S, Proud D (2007). Interleukin-17A modulates human airway epithelial responses to human rhinovirus infection.. Am J Physiol Cell Mol Physiol.

[21] Spira A, Beane J, Shah V, Liu G, Schembri F (2004). Effects of cigarette smoke on the human airway epithelial cell transcriptome.. Proc Natl Acad Sci U S A.

[22] Gelbman BD, Heguy A, O’Connor TP, Zabner J, Crystal RG (2007). Upregulation of pirin expression by chronic cigarette smoking is associated with bronchial epithelial cell apoptosis.. Respir Res.

[23] Pickett G, Seagrave J, Boggs S, Polzin G, Richter P (2010). Effects of 10 cigarette smoke condensates on primary human airway epithelial cells by comparative gene and cytokine expression studies.. Toxicol Sci.

[24] Spurrell JCL, Wiehler S, Zaheer RS, Sanders SP, Proud D (2005). Human airway epithelial cells produce IP-10 (CXCL10) in vitro and in vivo upon rhinovirus infection.. Am J Physiol Lung Cell Mol Physiol.

[25] Teran LM, Seminario MC, Shute JK, Papi A, Compton SJ (1999). RANTES, macrophage-inhibitory protein 1α, and the eosinophil product major basic protein are released into upper respiratory secretions during virus-induced asthma exacerbations in children.. J Infect Dis.

[26] Dufour JH, Dziejman M, Liu MT, Leung JH, Lane TE (2002). IFN-γ-inducible protein 10 (IP-10; CXCL10)-deficient mice reveal a role for IP-10 in effector T-cell generation and trafficking.. J Immunol.

[27] Trifilo MJ, Montalto-Morrison C, Stiles LN, Hurst KR, Hardison JL (2004). CXC chemokine ligand 10 control viral infection in the central nervous system: evidence for a role in innate immune response through recruitment and activation of natural killer cells.. J Virol.

[28] Nickel R, Beck LA, Stellato C, Schleimer RP (1999). Chemokines and allergic disease.. J Allergy Clin Immunol.

[29] Nakhaei P, Genin P, Civas A, Hiscott J (2009). RIG-I-like receptors: Sensing and responding to RNA virus infection.. Semin Immunol.

[30] Skaug B, Chen ZJ (2010). Emerging role of ISG15 in antiviral immunity.. Cell.

[31] Fensterl V, Sen GC (2011). The ISG56/IFIT1 gene family.. J Interferon Cytokine Res.

[32] Wark PAB, Johnston SL, Bucchieri F, Powell R, Puddicombe S (2005). Asthmatic bronchial epithelial cells have a deficient innate immune response to infection with rhinovirus.. J Exp Med.

[33] Contoli M, Message SD, Laza-Stanca V, Edwards MR, Wark PAB (2006). Role of deficient type-III interferon-λ production in asthma exacerbations.. Nature Medicine.

[34] Mallia P, Message SD, Gielen V, Contoli M, Gray K (2011). Experimental rhinovirus infection as a human model of chronic obstructive pulmonary disease exacerbation.. Am J Respir Crit Care Med.

[35] Lopez-Souza N, Favoreto S, Wong H, Ward T, Yagi S (2009). In vitro susceptibility to rhinovirus infection is greater for bronchial than for nasal airway epithelial cells in human subjects.. J Allergy Clin Immunol.

[36] Schneider D, Ganesan S, Comstock AT, Meldrum CA, Mahidhara R (2010). Increased cytokine response of rhinovirus-infected airway epithelial cells in chronic obstructive pulmonary disease.. Am J Respir Crit Care Med.

[37] Khaitov MR, Laza-Stanca V, Edwards MR, Walton RP, Rohde G (2009). Respiratory virus induction of alpha-, beta, and lambda-interferons in bronchial epithelial cells and peripheral blood mononuclear cells.. Allergy.

[38] Schmid S, Mordstein M, Kochs G, García-Sastre A, tenOever BR (2010). Transcription factor redundancy ensures induction of the antiviral state.. J Biol Chem.

